# Design of Cavity-Backed Bow-Tie Antenna with Matching Layer for Human Body Application

**DOI:** 10.3390/s19184015

**Published:** 2019-09-17

**Authors:** Jinho Jeong, Kihoon Park, Changmin Lee

**Affiliations:** Department of Electronic Engineering, Sogang University, 35 Baekbeom-ro, Mapo-gu, Seoul 04107, Korea

**Keywords:** antenna, bow-tie, cavity, internal body temperature, microwave, radiometer

## Abstract

This paper presents the broadband antenna for the microwave radiometric sensing of internal body temperature. For broadband operation, the bow-tie antenna was designed and backed with a cylindrical cavity, which decreased environmental electromagnetic interference and also improved the directivity of the antenna. The broadband impedance-transforming balun in microstrip form was also designed to feed the bow-tie antenna, and was located inside the cavity. An impedance-matching dielectric layer (IMDL) was introduced on top of the bow-tie antenna, for impedance match with the human body with high permittivity. The fabricated antenna was measured in free space with the IMDL removed, showing an input reflection coefficient lower than −10 dB from 2.64 to > 3.60 GHz with antenna gain over 6.0 dBi and radiation efficiency over 74.7% from 2.7 to 3.5 GHz. The IMDL was re-installed on the cavity-backed bow-tie antenna to measure the antenna performance for the human head with relative permittivity of about 40. The measured reflection coefficient was as low as −28.9 dB at 2.95 GHz and lower than −10 dB from 2.65 to > 3.5 GHz. It was also shown that the designed antenna recovered a good impedance match by adjusting the permittivity and thickness of the IMDL for the different parts of the human body with different permittivities.

## 1. Introduction

Microwave frequencies in the low GHz range allow good penetration of human tissues and high image resolutions. Therefore, there have been active researches in microwave applications for medical diagnosis and treatment [1], including the non-invasive detection of breast cancer and brain stroke based on the principle of the wideband radar or microwave tomography [2,3]. For example, ultra-wideband (UWB) microwave signals are illuminated in the human body, and the reflected waves are collected by broadband antenna to image and detect malignant tissues in breast cancer detection [2]. Microwaves have also been used for hyperthermia, where they generate heat inside the human body to destroy malignant tissues, such as cancer [4]. In addition, various microwave medical radiometers have been introduced to non-invasively measure and image internal body temperatures [5,6,7,8,9]. The on-body radiometric sensors can be used for the monitoring of core body temperature and deep brain temperature, and the detection of breast cancer [6,7,8,9,10]. 

An antenna is one of the key components in microwave medical applications. For on-body applications, where the antenna is in direct contact with, or in close proximity with, the body, the antenna should be designed to have an impedance match to the human body with high permittivity [1,10]. The antenna should also be adaptive to the wide variation of permittivity of human tissues. For medical radiometers, the broadband antenna is preferred to collect as much noise power radiated by the internal body [10], and should also suppress the interference signals from the environment to reduce radiometric sensing errors [11]. Mechanical flexibility is also a useful property of the on-body antenna in wearable sensor applications [12,13].

Several radiometric antennas have been introduced in the literature. In [6], a non-contact radiometer, using the cavity-backed slot antenna, was introduced at 1.4 GHz to monitor the core body temperature. A wideband log-spiral antenna was designed for deep-brain temperature sensing [10]; a three-frequency probe (near-field antenna) (1.4, 2.7, and 4.9 GHz) was fabricated in microstrip dipole form for wearable radiometers, to monitor the internal temperature of the human abdomen [7]. In [8], a cavity-backed elliptical antenna was introduced at 3.5 GHz, where the cavity was used as a vacuum chamber to provide suction for tight contact between the antenna surface and the body. Most of the reported radiometric antennas were mainly designed to broaden the impedance-matching bandwidth. 

In this paper, we present the cavity-backed bow-tie antenna with broadband impedance- matching with the human body, high directivity, and good shielding capability for the radiometric sensing of internal body temperature. For this purpose, the bow-tie patch was adopted as a basic radiating element with broadband performance. The cylindrical cavity was employed to suppress environmental interferences and to increase the directivity. The impedance-transforming balun was designed in microstrip form to feed the bow-tie antenna. In addition, the impedance-matching dielectric layer (IMDL) was introduced on top of the bow-tie patch, to reduce the wave reflections from the human body with high permittivity, and also to allow physical and thermal isolation of the antenna from the human body. Section 2 discusses the design of the bow-tie antenna, including the cavity, balun, and IMDL with 3-dimensional (3-D) electromagnetic (EM) simulation results. The measurement results of the fabricated antenna in free space and on-body contact mode are presented in Section 3. 

## 2. Design of the Cavity-Backed Bow-Tie Antenna

Human tissue exhibits a very high and wide range of relative permittivity ($\varepsilon_{r}$) from about 9 for fat and about 59 for blood [1,6]. A huge impedance mismatch must exist between the antenna and the human body if the antenna is designed to radiate into the air. Therefore, the antenna should be designed to consider the high $\varepsilon_{r}$ of the human body. First, we designed the bow-tie antenna radiating into the air, which was shielded by the metallic cavity. Then, the dielectric layer was introduced on top of the bow-tie antenna to match the impedance between the antenna and the human body.

### 2.1. Cavity-Backed Bow-Tie Antenna 

**Figure 1:**
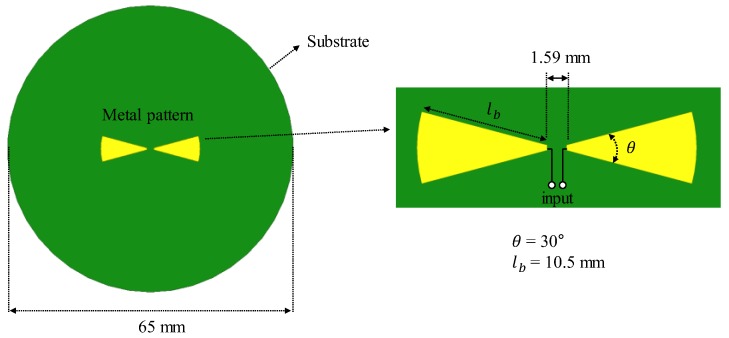
Designed bow-tie antenna.

The captured noise power by the radiometer is proportional to its operating bandwidth, which is determined by the antenna and filters. Therefore, the broadband antenna is usually adopted in medical radiometers. In this work, a bow-tie antenna was employed to achieve the broadband performance, as shown in Figure 1 [14]. The antenna was designed in 1.59 mm-thick substrate (CER-10 by Taconic) with a gold-plated 17 μm-thick copper layer. The substrate had a high $\varepsilon_{r}$ of 10.2, which was helpful in reducing the size of the bow-tie patch. The flare angle (θ) and length ($l_{b}$) of the bow-tie patch determined the input impedance and operating frequency of the antenna, respectively [15]; their values were determined as θ = 30° and $l_{b}$ = 10.5 mm by the high-frequency structure simulator (HFSS by Ansys) for broadband performance at the center frequency of 3.0 GHz. 

**Figure 2:**
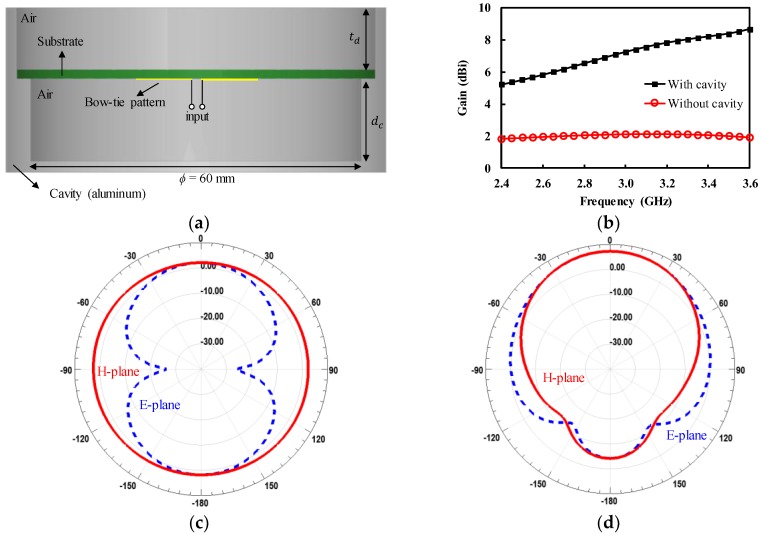
Cavity-backed bow-tie antenna (**a**) cross-sectional view; (**b**) simulated gain of the antenna with and without the cavity; (**c**) simulated gain of the antenna without the cavity; (**d**) simulated gain of the antenna with the cavity.

The designed bow-tie antenna is omni-directional, meaning it can receive the EM fields equally from the forward and backward directions; this implies that the antenna can collect interference signals from the environment besides the human body, which degrades the accuracy of the radiometers. Therefore, the antenna should be directive and shielded from environmental noise sources. The directive antenna is also useful for high spatial image resolution in the measurement of internal body temperature. In order to shield the bow-tie antenna from external noise sources and increase the directivity, the bow-tie antenna was backed by cylindrical cavity with a diameter of 60 mm, as shown in Figure 2a. If the bow-tie patch was placed about a quarter-wave length apart from the cavity walls, the backside radiation added in-phase with the frontside radiation after being reflected from the cavity walls, which led to an increase in the antenna directivity. The optimum distance ($d_{c}$) between the bow-tie patch and the bottom of the cavity was determined by the EM simulations to be 15.0 mm. The designed cavity increased the antenna gain from 2.1 to 6.9 dBi at 3 GHz, as shown in Figure 2b. Figure 2c,d show the simulated gain of the antenna without and with cavity, respectively. The designed cavity improved the front-to-back power ratio of 11.1 dB, which reduced the effect of environmental interferences. 

**Figure 3:**
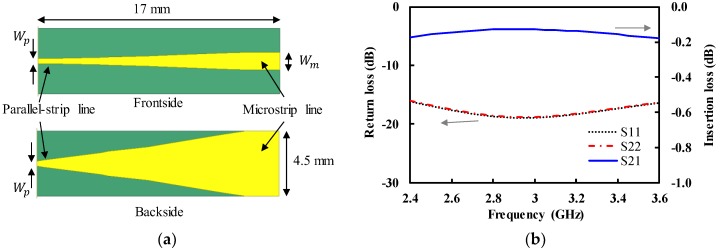
The designed impedance-transforming balun (**a**) layout; (**b**) simulated performance.

The bow-tie antenna was fed by the differential or balanced input signals. In addition, the input impedance of the cavity-backed bow-tie antenna in Figure 2a was as high as 120 Ω. Therefore, the impedance-transforming balun was designed using the same substrate as that of the bow-tie antenna, as shown in Figure 3a [16,17]. The balanced parallel strip line was connected to the input port of the bow-tie antenna. Both the front and backside metal patterns of the parallel strip line (width ($W_{p}$) = 0.34 mm) were tapered in order to gradually transform the impedance from 120 to 50 Ω in the microstrip line (width ($W_{m}$) = 1.2 mm). The tapered shape of the metal patterns was carefully determined to provide the broadband impedance-matching performance. The length was fixed by the distance ($d_{c}$) between the bow-tie patch and cavity, and the thickness of the cavity. Figure 3b shows the simulated insertion and return loss of the designed balun. The insertion loss was less than 0.2 dB with the return loss greater than 15 dB from 2.4 to 3.6 GHz. 

**Figure 4:**
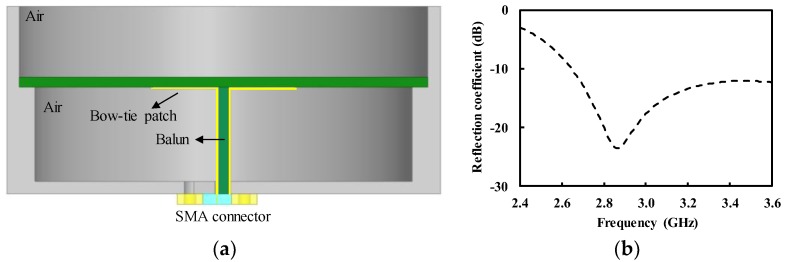
Cavity-backed bow-tie antenna with balun (**a**) cross-sectional view; (**b**) simulated reflection coefficient.

Figure 4a shows the cavity-backed antenna assembled with the balun and Sub-miniature version A (SMA) connector. The bow-tie patch was directed downward, so that it could be connected to the parallel strip line of the balun. The hole was drilled at the center of the bottom of the cavity, so that the microstrip line of the balun could be connected to the external SMA connector. Figure 4b shows the simulated reflection coefficient of the antenna (without the SMA connector) exhibiting a peak value of −23.5 dB at 2.87 GHz with the value lower than −10 dB across the very wideband from 2.65 to 3.60 GHz. 

### 2.2. Design of Impedance-Matching Dielectric Layer

**Figure 5:**
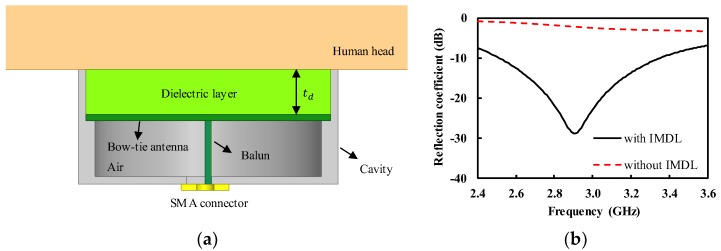
Cavity-backed bow-tie antenna for a human head (**a**) cross-sectional view; (**b**) simulated reflection coefficient.

The designed antenna in Section 2.1 was impedance-matched to that of the air, so that it would cause severe impedance-mismatch for the human body with high permittivity; this implies that most of the power radiated by the human body can be reflected from the antenna, which is not desirable for microwave radiometers. Therefore, we introduced the IMDL with a quarter-wave thickness, which was placed on the top of the bow-tie antenna, as shown in Figure 5a. We hypothesized the human head as a target, so that the designed antenna can be potentially used to measure brain temperature. The human head consists of several layers of different tissues, such as skin, fat, skull, and brain [10]. Each tissue exhibits different values in permittivity and conductivity, which are also frequency dependent [1,18]. Based on the data in the literature, we assumed the average relative permittivity and conductivity of the head as 40 and 2 S/m around 3 GHz. 

The wave impedance of the air and the head was calculated from $\eta =\sqrt{\mu /\varepsilon }$ to be 120 π and 59.6 Ω, respectively. Therefore, the quarter-wave long impedance-transformer, with a wave impedance of 250 Ω, was used to match the impedance between the air and the head, which corresponded to the dielectric layer with $\varepsilon_{r}$ of 6.3 and thickness ($t_{d}$) of 9.96 mm. In this work, the IMDL was fabricated by stacking several rubber sheets with relative permittivity of around 7; this also allowed the physical and thermal isolation of the antenna to the human body [19]. The $t_{d}$ was determined as 11.25 mm by 3-D EM simulation. Figure 5b shows the simulated reflection coefficient of the antenna of Figure 5a. The peak reflection coefficient of −28.8 dB was obtained at 2.91 GHz for the head, thanks to the IMDL. The IMDL allowed the broadband impedance match to the head, with reflection coefficient of less than −10 dB from 2.52 to 3.35 GHz. On the contrary, the reflection coefficient was higher than −3.1 dB from 2.4 to 3.6 GHz if the IMDL was removed, indicating a severe impedance mismatch. 

**Figure 6:**
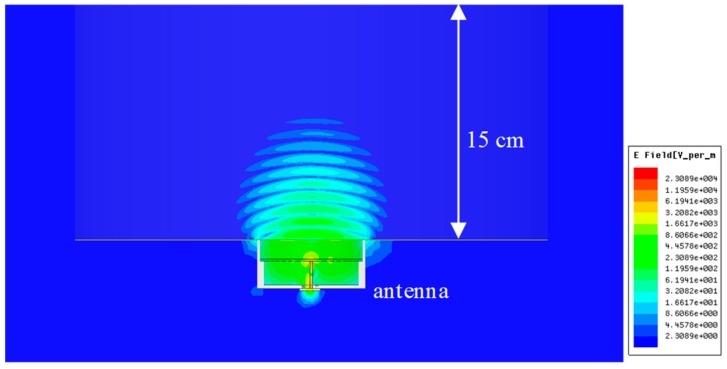
Simulation of electric field distribution inside the human body.

Figure 6 shows the simulated electric field distribution inside the head phantom ($\varepsilon_{r}$ = 40 and conductivity (σ) = 2.0 S/m) at 3.0 GHz, demonstrating that the electric field intensity can penetrate into the body with high directivity. The power intensity reduced to one-tenth of that on the surface at the distance of 2.5 cm from the surface. 

## 3. Fabrication and Measurement

### 3.1. Fabrication

**Figure 7:**
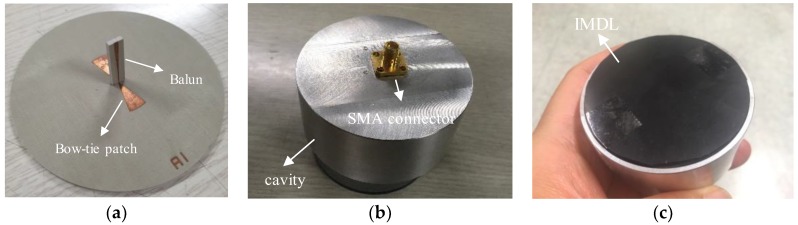
Fabricated antenna (**a**) bow-tie antenna and balun; (**b**) cavity with SMA connector; (**c**) impedance-matching dielectric layer on top of the bow-tie substrate.

The designed antenna was fabricated, as shown in Figure 7. The two inputs of the bow-tie antenna were soldered to the front and backside metal patterns of the balun, as shown in Figure 7a. Then, the balun substrate was mounted on the hole in the bottom of the cavity and connected to a SMA connector, as shown in Figure 7b. Finally, the upside of the bow-tie substrate was covered by the butadiene rubber (IMDL), as shown in Figure 7c. 

### 3.2. Measurement of the Antenna in Free Space

**Figure 8:**
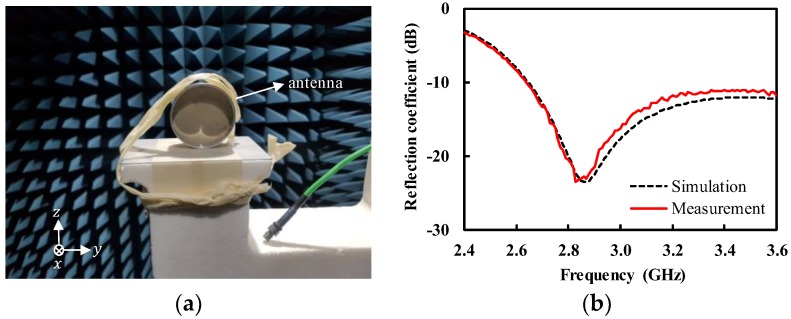
Measurement of the antenna in free space (with the impedance-matching dielectric layer (IMDL) removed) (**a**) antenna in the anechoic chamber; (**b**) reflection coefficient.

**Figure 9:**
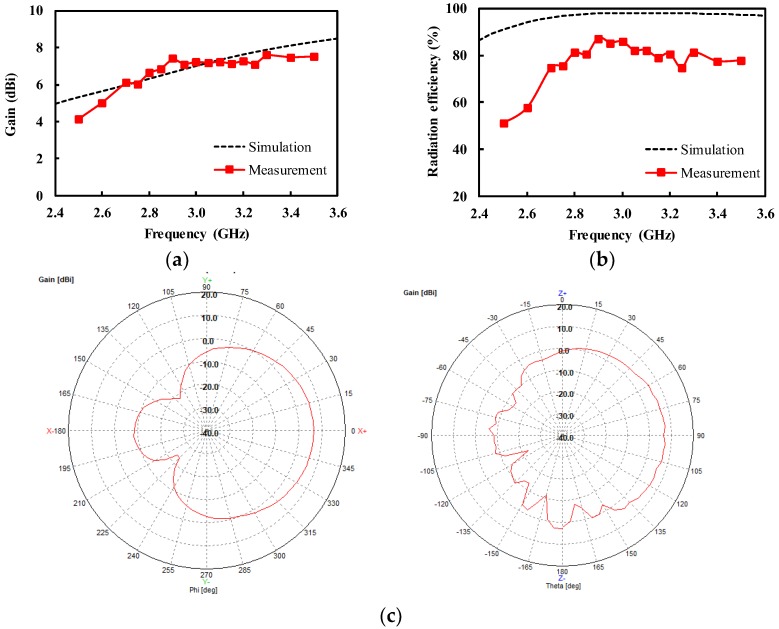
Measurement of the antenna in free space (with the IMDL removed) (**a**) antenna gain; (**b**) radiation efficiency; (**c**) measured radiation pattern at 3.0 GHz (left: E-plane, right: H-plane).

Prior to measuring the fabricated antenna in contact with the human body, we performed the measurement in free space to characterize the antenna performance, such as radiation pattern, antenna gain, and radiation efficiency. Figure 8a shows the measurement of the fabricated antenna in the anechoic chamber. The performance of the designed antenna was measured using a vector network analyzer and waveguide horn antenna. The free-space measurement was performed with the IMDL removed. The measured reflection coefficient exhibited a minimum value of −23.5 dB at 2.83 GHz, lower than −10 dB from 2.64 to > 3.60 GHz, as shown in Figure 8b. The reflection coefficient showed a close agreement between simulation and measurement. Figure 9a shows the measured antenna gain with a peak of 7.4 dBi at 2.9 GHz. The measured antenna gain and radiation efficiency were higher than 6.0 dBi and 74.7% (Figure 9b), respectively, from 2.7 to 3.5 GHz. There was a somewhat large discrepancy between the simulation and measurement of the radiation efficiency. It is known that the numerical simulation often over-estimates the radiation efficiency [20,21]. The radiation efficiency in the measurement was obtained from the relationship between the gain and directivity, whereas the simulated one was based on the ratio of the total radiated power to the accepted power by the antenna. The radiation pattern was also measured at 3.0 GHz, as shown in Figure 9c. The front-to-back power ratio was as high as 15.1 dB, thanks to the shielding cavity.

### 3.3. Measurement of the Antenna on the Human Body

**Figure 10:**
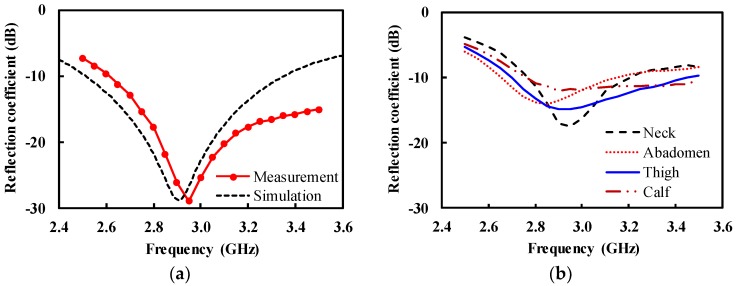
Measurement of the antenna on the human body (**a**) head; (**b**) various human parts (neck, abdomen, thigh, and calf).

For on-body measurement, the IMDL was re-installed on the antenna. Then, the antenna was placed in contact with the human body. Figure 10a shows the measured input reflection coefficient for the head, with a minimum value of −28.9 dB at 2.95 GHz and the value lower than −10 dB from 2.65 to 3.5 GHz. The measurement shows a good agreement with the simulation, except for the center frequency which was about 50 MHz higher than the simulation. The same antenna was used to measure the reflection coefficient for the various parts of the human body, as shown in Figure 10b. The reflection coefficient was seriously degraded with the bandwidth reduced, compared with the case for the head; this is because the permittivity varied depending on the constituting tissues of the human body.

**Figure 11:**
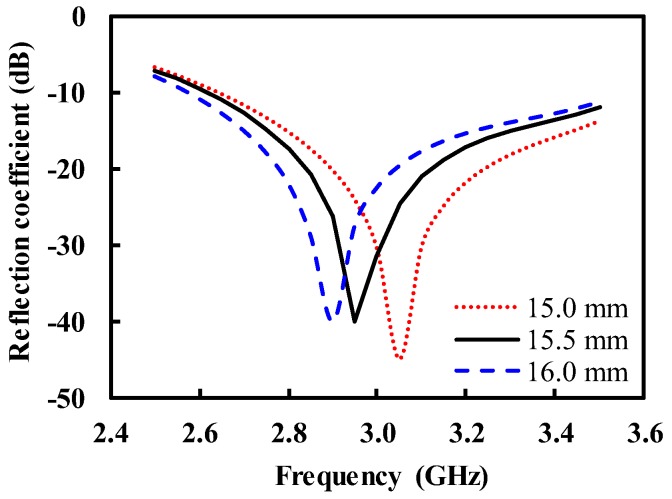
Simulated reflection coefficient for the human body with $\varepsilon_{r}$ = 20 as a function of the thickness ($t_{d}$) of the IMDL with $\varepsilon_{r}$ = 4.5.

However, it can be shown from the simulation that the impedance match can be recovered by adjusting the permittivity and thickness of the IMDL, depending on the permittivity of the human body. For example, the modified IMDL with $\varepsilon_{r}$ of 4.5 and $t_{d}$ of 15.5 mm restored a good impedance match at 3.0 GHz and broad bandwidth for the human body with $\varepsilon_{r}$ of 20, as shown in Figure 11.

## 4. Conclusions

In this paper, the broadband antenna for microwave medical applications was designed using the cavity-backed bow-tie antenna. The cavity was introduced to reject environmental interference signals, as well as to increase the directivity. The impedance-transforming balun was designed to connect the balanced inputs of the bow-tie antenna and the SMA connector. The impedance-matching dielectric layer was proposed and stacked on top of the bow-tie antenna, to effectively reduce the reflections between the antenna and the human body. The designed antenna exhibited broadband impedance-matching performance with high gain and efficiency in the free-space measurement. The on-body measurement of the antenna also showed broadband-matching performance. It was shown from the simulation that the antenna maintained a good impedance match for various parts of the human body with different permittivities by modifying the impedance-matching dielectric layers. Therefore, we believe that the designed antenna can be successfully applied for the radiometric sensing of internal body temperature, as well as microwave medical diagnosis and treatment.
